# Host Delivery of Favorite Meals for Intracellular Pathogens

**DOI:** 10.1371/journal.ppat.1004866

**Published:** 2015-06-25

**Authors:** Yousef Abu Kwaik, Dirk Bumann

**Affiliations:** 1 Department of Microbiology and Immunology, Center for Predictive Medicine, College of Medicine, University of Louisville, Louisville, Kentucky, United States of America; 2 Focal Area Infection Biology, Biozentrum, Universität Basel, Basel, Switzerland; University of North Carolina at Chapel Hill School of Medicine, UNITED STATES

## Introduction

Pathogens grow and cause disease by exploiting the host as a rich and diverse source of food. However, it is not always an easy task to tap these food resources since the host innate immune response restricts pathogen access to crucial nutrients (“nutritional immunity”) [1]. Pathogens have acquired various mechanisms to evade host nutritional innate immunity and to trigger the host to generate additional preferable sources of carbon, nitrogen, and energy.

Pathogens utilize various nutrients at vastly different rates. Some nutrients such as metal ions, cofactors, and monomeric components of proteins, lipids, and carbohydrates are directly incorporated into biomass. In addition, pathogens need to degrade substantial amounts of nutrients to small excreted waste products in order to obtain the energy they require for assembling biomass components and maintaining homeostasis (such as counteracting dissipation of membrane gradients). Uptake and metabolism of such energy sources is generally much faster compared to nutrients that are directly incorporated into new biomass.

While extracellular pathogens can often exploit rich energy sources delivered to them by the host circulation, intracellular bacterial pathogens depend on their surrounding host cells for supply of energy sources at sufficiently high rates. This extensive metabolic interplay between host cells and the pathogens that they nurture is likely full of fascinating, rich biology. However, these major fluxes remain poorly characterized since common methods to study pathogen metabolism such as tracking incorporation of isotope-labelled carbon/nitrogen into biomass are not informative on nutrients converted into excreted waste products.

On the other hand, new approaches start to unravel how intracellular pathogens acquire energy sources at sufficiently high rates for growth and disease—in particular, intravacuolar pathogens that must import nutrients across the vacuolar membrane. This Pearl article will highlight acquisition of energy sources by intravacuolar pathogens and its role in disease. For other aspects of microbial nutrition in vivo and host mechanisms for nutrient restriction, the reader is referred to various recent reviews [2–5].

## The Hunger for Energy

Bacterial proliferation requires high amounts of energy. For bacteria such as *Escherichia coli*, generating a daughter cell requires hydrolysis of some 8 x 10^9^ adenosine triphosphate (ATP) molecules to assemble biomass and support essential maintenance requirements, even if all monomeric components (amino acids, nucleosides, sugars, etc.) are freely available [6]. Even in minimal media with a single carbon source such as glucose where the bacterium has to synthesize all biomass components itself, energy production is the single most important metabolic activity of *E*. *coli* (about 37% of glucose is used for ATP generation) [6]). Pathogens thus must access a suitable host energy source to cause disease. Relevant energy sources can be identified based on consumption and waste product profiles as measured by metabolomics or from major growth defects of strains defective for certain nutrient utilization pathways.

For extracellular pathogens with direct access to blood or interstitial fluid, host glucose and glutamine provide rich energy sources that are rapidly replenished by the host circulation. In contrast, intracellular pathogens access diverse host cell metabolites, but these nutrients are quickly exhausted if not actively replenished by the host cell. As an example, ten *Shigella* cells that rapidly grow in the cytosol of a human epithelial cell would completely consume the most abundant host metabolites within just a few minutes [6,7]). Therefore, a robust continuous host nutrient supply pipeline within viable host cells is essential to meet the energy demands of intravacuolar pathogens that also face the challenge of importing across the pathogen-containing vacuole.

**Fig 1:**
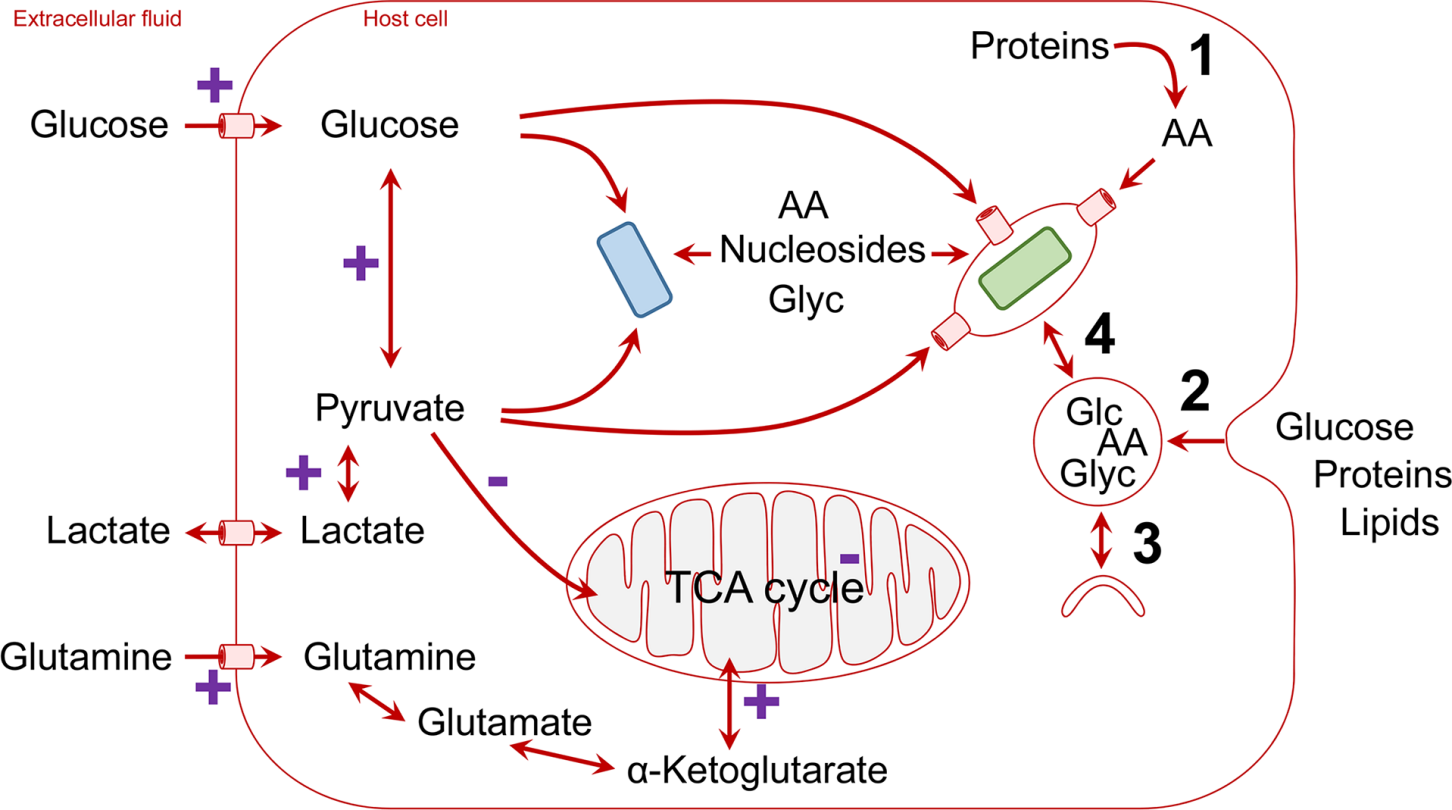
Schematic overview of host nutrient supply for intracellular pathogens (blue, cytosolic pathogen; green, vacuolar pathogen; AA, amino acids; Glc, glucose; Glyc, glycerol). Cellular mechanisms that convert polymeric nutrients into small building blocks and deliver them to vacuolar pathogens are shown on the right (1, degradation of proteins to amino acids by proteasomes; 2, endocytosis and degradation in lysosomes; 3, autophagosome formation and delivery to lysosomes; 4, vesicle trafficking and fusion/luminal exchange with pathogen-containing vacuole). Pathways that are stimulated (+) or repressed during hypoxic conditions within inflammatory foci are labelled in purple.

What nutrients can be delivered by host cells at sufficiently high rates to meet the energy demands of intracellular pathogens? The host cells mostly depend on abundant blood metabolites, in particular glucose and glutamine, but also lactate in areas with limited oxygenation (Fig 1). Host cells possess high-rate uptake systems for these metabolites, and during inflammation, glucose transport is even further enhanced.

Pathogens utilize diverse sources of host energy (Table 1). Cytosolic pathogens such as enteroinvasive *E*. *coli* (EIEC) can directly use incoming glucose (Fig 1) [2], whereas intravacuolar pathogens can access host cell glucose when using pathogen-encoded or host cell glucose transporters in the vacuolar membrane (Fig 1) [8]. As an alternative pathway, *Salmonella*-containing vacuoles have extensive exchange with endocytic vesicles [9,10], which may enhance acquisition of glucose from the extracellular environment [2,11]. Interestingly, activation of peroxisome proliferator-activated receptor γ (PPAR γ) or PPARδ enhances glucose availability for intravacuolar pathogens in the M2 subset of host macrophages and promotes pathogen persistence, which has been shown for *Salmonella* and *Brucella* [12,13]. However, during acute infection, glucose in infected tissues seems to play a moderate role for *Salmonella* nutrition [14].These studies show a clear dynamic interaction between the host metabolism and metabolism of intravacuolar pathogens.

**Table 1:** Main energy sources of intracellular pathogens.

Pathogen	Intracellular Localization	Main Energy Source	Supply Route	Reference
Enteroinvasive *E*. *coli*	cytosol	glucose	host cell uptake	[2]
*Shigella flexneri*	cytosol	pyruvate	host cell glycolysis/host uptake	[7]
*Listeria monocytogenes*	cytosol	glycerol	glucose conversion	[26]
*Plasmodium*	vacuole	glucose	host cell uptake	[8]
*Salmonella enterica*	vacuole	diverse nutrients with glycerol and fatty acids as major sources of energy during acute infection, glucose during persistence	?	[10,14]
*Brucella*	vacuole	glucose	?	[11]
*Anaplasma phagocytophilum*	vacuole	amino acids	autophagy	[21]
*Legionella pneumophila*	vacuole	amino acids	proteasome	[22]
*Mycobacterium tuberculosis*	vacuole	cholesterol	?	[27]

Lactate is excreted by cells that ferment glucose and can be reimported by other cells. Fermentation occurs in tissues with limited oxygen supply [15], a condition often encountered in infected tissues. In addition, activated macrophages also switch from respiration to fermentation, even in the presence of ample oxygen [16]. As a result, lactate levels are elevated in many infected host microenvironments. Extracellular lactate can be rapidly imported by cells and is immediately converted in the cytosol into pyruvate if enough oxygen is present to consume released reduction equivalents (Fig 1). An alternative route to pyruvate is host cell glucose uptake and metabolism through glycolysis. Being one of the major high-flux pathways in mammalian cells, glycolysis can continuously provide pyruvate at very high rates that easily meet even voracious demands for fast-growing intracellular *Shigella* (Fig 1) [7]. Various other intracellular pathogens, such as *Legionella*, can also effectively utilize pyruvate, but its mechanism of import into the pathogen-containing vacuoles and whether it plays a role in nutritional virulence are not yet established [7,17–19].

It is important to note that in primary cells with sufficient oxygenation, host cell mitochondria also take up pyruvate for fueling the tricarboxylic acid (TCA) cycle. Pathogens will thus compete with the mitochondria for the cytoplasmic pyruvate pool (Fig 1). This important aspect might be underestimated in common cell culture infection models that employ cell lines with minor mitochondrial pyruvate uptake even in presence of oxygen (aerobic glycolysis, the Warburg effect; see below). On the other hand, even fully functional mitochondria have a pyruvate transporter with only moderate affinity (K_M_ in the range of 0.5 mM) and low transport rate [20], compared to bacteria such as *E*. *coli*, which has at least two high-affinity pyruvate transporters (K_M_ in the range of 10 μM) [21]. Pathogens might thus effectively compete with mitochondria even in primary cells with active respiration.

In addition to exploiting host cell nutrient uptake, or host metabolism, some intravacuolar pathogens employ diverse sophisticated mechanisms to exploit valuable nutrients released by host cell degradation of polymeric biomass components, in particular proteins (Fig 1). *Coxiella* resides in phagolysosomes, where it resists the highly adverse conditions and captures amino acids released from proteins as part of the normal host cell protein turnover [22]. *Anaplasma phagocytophilum* uses the type IV-translocated effector 1 (Ats-1) to promote the host autophagy degradation pathway and gain access to amino acids [23]. In contrast, *Legionella pneumophila* also use amino acids (or amino acid-derived pyruvate) as the main intracellular energy sources that are metabolized in their TCA cycle [24]. To mobilize sufficient levels of host cell amino acids, *Legionella* injects into the host cell the type IV-translocated Ankyrin B (AnkB) effector, which functions on the pathogen-containing vacuole (PCV) as a platform for the assembly of polyubiquitinated proteins, which are targeted for proteasomal degradation [17]. Inhibition of AnkB-dependent proteasomal degradation blocks *Legionella* growth within the PCV, and this growth defect is totally bypassed upon supplementation of cysteine (Cys), serine (Ser), alanine (Ala), pyruvate, or citrate, all of which feed the TCA cycle [17].

Importantly, diverse sources of nutrients are most likely captured within a specific tissue as major sources of carbon and energy (Table 1). *Salmonella* access many diverse host nutrients in infected mouse spleen [14]. Major nutrients include glycerol and fatty acids that are presumably released by lipid degradation. In addition, *Salmonella* obtains carbohydrates such as N-acetylglucosamine [14], which is usually part of macromolecules, suggesting again host cell degradation as part of the nutrient supply pipeline. Together, data for *Salmonella* suggest that instead of one major energy source, the host–pathogen metabolic interface can be much more complex, with a diversified portfolio of energy sources. Interestingly, glycerol generated from lipid degradation and/or glycolytic intermediates can also be a major energy source for intracellular pathogens (Table 1) [3,25,26]. The intravacuolar pathogen *Mycobacterium tuberculosis* seems to be peculiar, as it mainly consumes host lipids such as cholesterol as sources of energy [27] but also remodels some of these lipids to generate its own essential lipids, including mycolic acids [28]. We speculate that it is more likely that many intravacuolar pathogens have evolved to utilize a diverse portfolio of host energy sources that are imported into the PCV lumen, instead of relying on one nutrient that may become scarce under certain conditions (Table 1).

## Pathogen Sources of Energy as Essential Host Metabolites

There is an emerging theme that many sources of energy, and amino acids in particular, are essential for intracellular pathogens as well as their host cells. Human cells are auxotrophic for nine amino acids (leucine [Leu], isoleucine [Ile], methionine [Met], valine [Val], threonine [Thr], phenylalanine [Phe], tryptophan [Trp], histidine [His], and lysine [Lys]), while Cys is semiessential and is the most limiting amino acid in human cells. Therefore, intracellular pathogens have evolved with nutritional strategies to enhance the level of these essential sources of energy. The cytosolic pathogen, *Francisella*, is auxotrophic for six amino acids (His, Lys, Met, Cys, arginine [Arg], and tyrosine [Tyr]). Interestingly, *Francisella* boosts the levels of free Cys in the host cell cytosol using its γ-glutamyl transpeptidase (Ggt) enzyme to cleave host glutathione (GSH) (L-γ-L-glutamyl-L-Cysteinyl-glycine) [29]. Similarly, the intravacuolar pathogen *Legionella* is auxotrophic for several amino acids (Leu, Ile, Met, Val, Thr, Cys, and Arg), five of which are essential for human cells [30]. We speculate that access of intracellular pathogens to host energy sources has been a major factor in nutritional evolution and adaptation of pathogens to the intravacuolar environment. Future studies should determine the role of host auxotrophy in the nutritional and metabolic evolution of intracellular pathogens [30,31].

## Importing Nutrients across the PCV Membrane

Intravacuolar pathogens are faced with the additional challenge of importing nutrients across the vacuolar membrane. However, there is very limited knowledge of how intravacuolar pathogens import nutrients from the host cell cytosol across the vacuolar membrane and into the lumen of the pathogen-containing vacuole (Fig 1). In addition to the transporters/pores employed by *Plasmodium* and *Toxoplasma* (see above), evidence for bacterial pathogens suggests participation of host solute-carrier (SLC) transporters, the second largest superfamily (~400 putative transporters) of membrane proteins in humans [32]. The SLCs include passive transporters, Na^+^- or H^+^-coupled symporters, and antiporters, located in cellular and organelle membranes. About 25% of all SLCs are members of seven SLC families that transport amino acids, but other substrates such as glucose, lipids, and drugs are also transported by specific SLCs.

In particular, it has been shown that the host cationic amino acid transporter SLC7A1 is acquired by the PCV-harboring *Salmonella* and *Mycobacterium* within macrophages, where it imports Arg across the pathogen-containing vacuolar membrane [33]. SLC1A5, which imports neutral amino acids, is essential for intravacuolar proliferation of *Legionella* [34], but it remains to be determined whether the transporter is localized to the membrane of the PCV (Fig 1). High-throughput proteomic analyses of the *Legionella* PCV within human macrophages indicated the presence of a few SLCs that transport various amino acids [35], and transcriptome analysis has shown up-regulation of many SLCs during infection [36]. In addition, some pathogens might translocate their own nutrient transporters to be incorporated into the vacuolar membrane (as proposed for the *Toxoplasma* pore). Finally, PCVs might extensively communicate with other cellular vesicles, exchanging luminal contents. This has been documented for *Salmonella*, which acquires extracellular nutrients through stealing cargo of normal host cell endocytosis [9,10].

## Modulation of Host Cell Metabolism during Infection In Vivo—Challenges

It is clear that energy supply is one of the most crucial aspects determining pathogen growth and virulence. However, still only a small minority of pathogen metabolism studies are focused on this important issue (Table 1). The classical focus on auxotrophic strains is not informative for energy production, and sophisticated metabolomic studies that determine carbon and/or nitrogen label incorporation in biomass can yield only indirect evidence for energy. The most direct approach to unravel pathogen energy production is quantitative analysis of metabolic waste products in combination with various labeling and mutagenesis strategies [7]. We can expect more insights from similar approaches for various pathogen–host interactions.

However, separation of host and pathogen metabolites to directly determine participation of host and pathogen pathways remains challenging because of the very short turnover time, which can result in substantially altered metabolomes during pathogen purification attempts. New techniques that can follow metabolites in a spatially resolved manner would offer fascinating opportunities to overcome these difficulties. In the meantime, specific perturbation of host and/or pathogen enzymes can provide important insights on relevant pathways and their localization in the host or pathogen cells. Once major energy sources have been unraveled, host pathways that supply them at sufficiently high rates can be investigated. In many cases, such pipelines are manipulated by the pathogen as a major part of their molecular virulence mechanisms.

Our knowledge of metabolic host responses to bacterial pathogens during infection is still limited because of the major experimental challenges of the infection model and the complicated analytical tools and methods. Many studies have utilized metabolic flux analysis revealing host cell metabolic alterations and carbon fluxes during infection by various pathogens (see [37] for a recent review). In general, some common themes have been observed, but otherwise there are major differences between the pathogens in terms of the host metabolic modulation during infection. Some of these modulations can be caused by direct manipulation of the host metabolism by the pathogen, while others are indirect host cell responses to infection. However, the in vitro tissue culture systems used to determine the host cell metabolic response are difficult to extrapolate to the in vivo conditions in infected tissues.

We focused this Pearl article on the diverse strategies utilized by various intracellular pathogens to acquire preferable energy sources at sufficiently high rates within the host cell. However, the two main carbon and nitrogen sources for mammalian cells are glucose (Glu) and glutamine (Gln), which are imported by SLC transporters [32]. A two-enzymatic step converts Gln into Glu and then to α-ketogluterate, which feeds the TCA cycle (Fig 1). In most differentiated cells, there is a balanced carbon flux through various catabolic pathways, while oxidative phosphorylation from the TCA cycle is the main route to generate ATP [37]. However, most transformed cells utilize glycolysis as the main catabolic pathway for generation of ATP, which has been designated as aerobic glycolysis or the Warburg effect [37]. In addition to enhanced glycolysis in transformed cells, glutaminolysis is enhanced, which provides TCA intermediates (Fig 1) [37]. Tissue culture studies using primary or transformed cell lines utilize media containing high levels of glucose and amino acids (especially glutamine) and growth factors, which alter cellular regulation of nutrient transporters and metabolic pathways. These in vitro nutritional environments are rarely encountered by bacterial pathogens in vivo. Therefore, the metabolic responses observed in tissue culture may vary considerably from the in vivo environment. Moreover, in response to hypoxia encountered within inflammatory foci, the cells respond to it through up-regulation of the hypoxia inducible factor (HIF-1) [38,39], which activates hundreds of genes required for adaption to hypoxia, including the glucose transporter and glycolytic enzymes as well as lactate dehydrogenase [5,40]. In addition, part of the inflammatory response is triggering nuclear factor kappa B (NF-κB), which is a major regulator of various nutrient transporters and metabolic pathways [5,40]. However, since macrophages in hypoxic inflammatory foci undergo metabolic shift to aerobic glycolysis and enhanced glutaminolysis, the generation of high levels of lactate and pyruvate and TCA intermediates [5,37,40] may provide a major source of carbon and energy for various intracellular pathogens in vivo (Fig 1). However, in vitro modeling of the dynamic hypoxic inflammatory foci that contain various host cells would be very challenging with the current technologies available. It is clear that major advances in the field will depend on the development of innovative tools and technologies to model the infection in vitro and to overcome the challenges in deciphering host-microbe nutritional and metabolic cross talk in vivo.
